# Organophosphorus Pesticides Decrease M2 Muscarinic Receptor Function in Guinea Pig Airway Nerves via Indirect Mechanisms

**DOI:** 10.1371/journal.pone.0010562

**Published:** 2010-05-10

**Authors:** Becky J. Proskocil, Donald A. Bruun, Charles M. Thompson, Allison D. Fryer, Pamela J. Lein

**Affiliations:** 1 Division of Pulmonary and Critical Care Medicine, Oregon Health & Science University, Portland, Oregon, United States of America; 2 Department of Molecular Biosciences, University of California Davis, Davis, California, United States of America; 3 Center for Structural and Functional Neuroscience, Department of Biomedical and Pharmaceutical Sciences, University of Montana, Missoula, Montana, United States of America; University of Pittsburgh, United States of America

## Abstract

**Background:**

Epidemiological studies link organophosphorus pesticide (OP) exposures to asthma, and we have shown that the OPs chlorpyrifos, diazinon and parathion cause airway hyperreactivity in guinea pigs 24 hr after a single subcutaneous injection. OP-induced airway hyperreactivity involves M2 muscarinic receptor dysfunction on airway nerves independent of acetylcholinesterase (AChE) inhibition, but how OPs inhibit neuronal M2 receptors in airways is not known. In the central nervous system, OPs interact directly with neurons to alter muscarinic receptor function or expression; therefore, in this study we tested whether the OP parathion or its oxon metabolite, paraoxon, might decrease M2 receptor function on peripheral neurons via similar direct mechanisms.

**Methodology/Principal Findings:**

Intravenous administration of paraoxon, but not parathion, caused acute frequency-dependent potentiation of vagally-induced bronchoconstriction and increased electrical field stimulation (EFS)-induced contractions in isolated trachea independent of AChE inhibition. However, paraoxon had no effect on vagally-induced bradycardia in intact guinea pigs or EFS-induced contractions in isolated ileum, suggesting mechanisms other than pharmacologic antagonism of M2 receptors. Paraoxon did not alter M2 receptor expression in cultured cells at the mRNA or protein level as determined by quantitative RT-PCR and radio-ligand binding assays, respectively. Additionally, a biotin-labeled fluorophosphonate, which was used as a probe to identify molecular targets phosphorylated by OPs, did not phosphorylate proteins in guinea pig cardiac membranes that were recognized by M2 receptor antibodies.

**Conclusions/Significance:**

These data indicate that neither direct pharmacologic antagonism nor downregulated expression of M2 receptors contributes to OP inhibition of M2 function in airway nerves, adding to the growing evidence of non-cholinergic mechanisms of OP neurotoxicity.

## Introduction

Asthma prevalence and severity has increased over the past two decades, with the greatest increase occurring in children and adolescents living in urban environments [1], [2]. Over this same period, the use of organophosphorus pesticides (OPs) has increased not only in agricultural environments [3]–[6] but also significantly in residential and urban settings [7]–[11]. Currently, OPs are the most widely used chemical pesticides in the United States and throughout the world [12], and are approved for a variety of commercial and household applications, including control of cockroach antigen [7]–[10], which is thought to be a primary trigger of asthma [13], [14]. Epidemiological and clinical studies have linked OP exposures to symptoms associated with asthma including airway hyperreactivity and wheezing [15]–[23]. Consistent with these findings from human studies, it has been reported that OPs induce bronchospasm in a variety of animals [24], [25], and we have recently established that the OPs chlorpyrifos, parathion and diazinon induce airway hyperreactivity in a guinea pig model [26], [27].

It is widely postulated that OPs trigger airway hyperreactivity via inhibition of acetylcholinesterase (AChE, E.C. 3.1.1.7) [28], [29], which decreases hydrolysis of acetylcholine (ACh) [29], [30] resulting in bronchoconstriction via prolonged activation of M3 muscarinic receptors on airway smooth muscle [31]–[33]. However, we observed that OPs potentiated vagally-induced bronchoconstriction in the guinea pig in the absence of AChE inhibition [26], [27]. Rather, the mechanism involved decreased function of autoinhibitory M2 muscarinic receptors on the parasympathetic nerves supplying airway smooth muscle [26], [27]. Activation of these M2 receptors functions as a negative feedback mechanism, decreasing ACh release from prejunctional parasympathetic nerves to limit vagally-induced bronchoconstriction [31], [34], [35]. Loss of M2 receptor function leads to increased ACh release from parasympathetic nerves resulting in potentiation of vagally mediated bronchoconstriction, which contributes to airway hyperreactivity. OP-induced inhibition of M2 receptor function in airway nerves and the consequent airway hyperreactivity is consistent with previous studies demonstrating that neuronal M2 receptors are dysfunctional in animal models of antigen-, virus- or ozone-induced airway hyperreactivity [36]–[38], and in patients with asthma [39].

What is not yet clear is how OPs decrease M2 receptor function in airway nerves. Studies of antigen-induced airway hyperreactivity have established a role for a specific inflammatory cell, the eosinophil, in causing M2 receptor dysfunction in airway nerves [40]–[43]. Similar studies of OP-induced airway hyperreactivity in guinea pigs revealed that the contribution of eosinophils to M2 dysfunction in animals exposed to the OP parathion varies with atopic status. Pre-treatment with an antibody to interleukin-5 to deplete eosinophils effectively prevented parathion-induced airway hyperreactivity in guinea pigs sensitized to ovalbumin protein, but had no effect in naïve animals [44], indicating that mechanism(s) independent of eosinophils underlie OP-induced M2 receptor dysfunction in non-atopic individuals.

Plausible alternative mechanism(s) by which OPs might cause neuronal M2 receptor dysfunction in airway nerves of non-atopic individuals include direct pharmacologic antagonism of M2 receptor function or direct interactions with neurons to downregulate M2 receptor expression. There are numerous reports documenting the ability of OPs to modulate muscarinic receptor function and/or expression in the brain independent of AChE inhibition (reviewed in [45], [46]). Whether similar interactions occur between OPs and peripheral neurons is not known, but that such interactions might be functionally significant in airway hyperreactivity is suggested by studies implicating decreased M2 receptor expression in virally induced airway hyperreactivity [47]–[49]. Therefore, in this study, we tested the hypothesis that the OP parathion, which we previously demonstrated causes airway hyperreactivity via inhibition of M2 receptor function in airway nerves [26], directly antagonizes M2 receptor function or downregulates M2 receptor expression in peripheral airway nerves. Our findings do not support this hypothesis, suggesting that OPs inhibit M2 receptor function in the airways of non-atopic individuals via indirect mechanisms involving a yet to be identified intermediary cell.

## Results

### Acute *in vivo* effects of parathion or paraoxon administered intravenously

**Figure 1:**
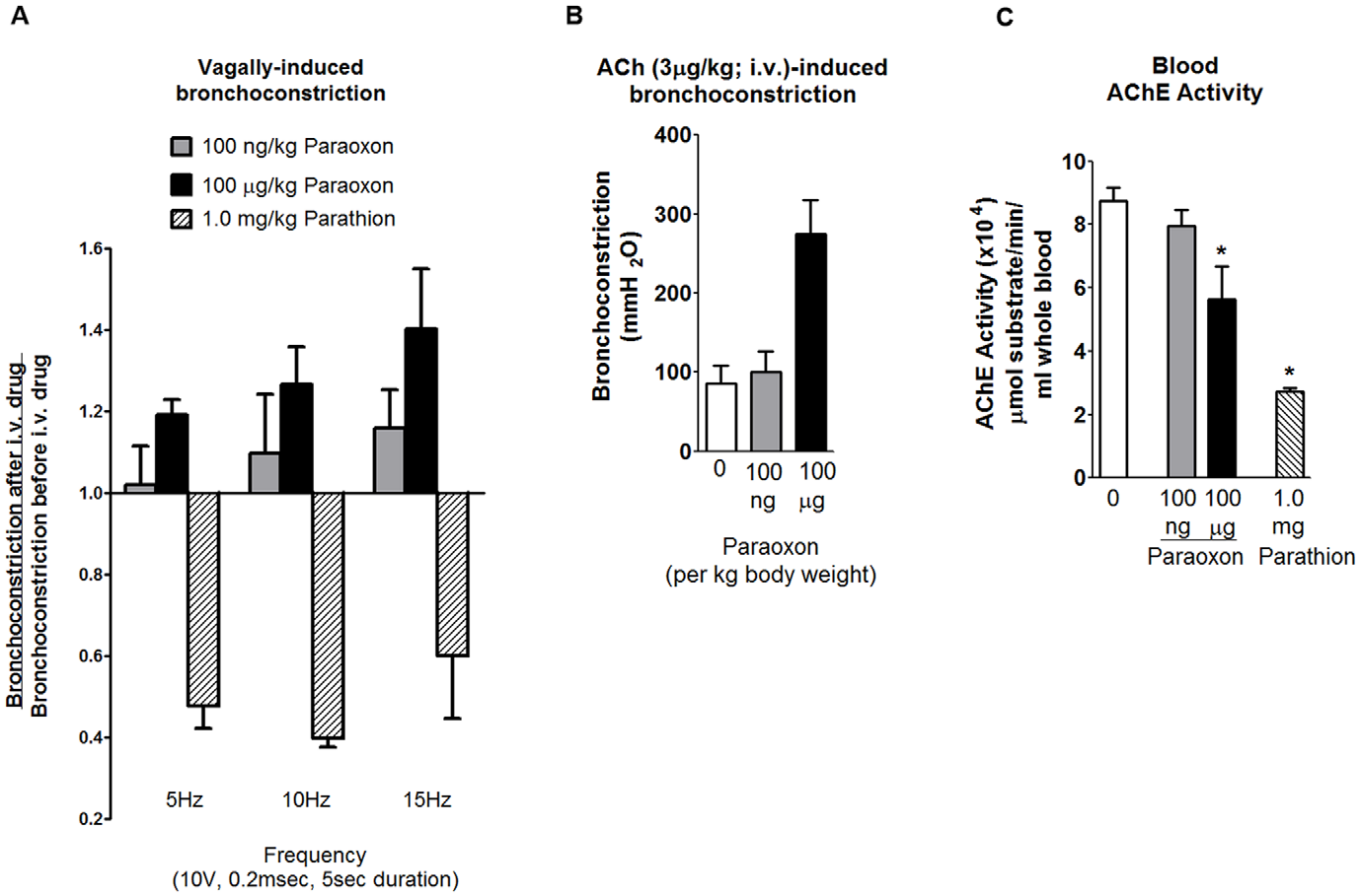
Effects of acute intravenous administration of parathion or paraoxon on bronchoconstriction in guinea pigs. Bronchoconstriction was measured in anesthetized guinea pigs in response to electrical stimulation of both vagus nerves or to intravenous ACh before and after intravenous administration of parathion (1 mg/kg) or paraoxon (100 ng/kg or 100 µg/kg). Baseline bronchoconstrictions were within normal physiological parameters (8–17 mmH2O at 5 Hz, 17–59 mmH2O at 10 Hz, and 51–127 mmH2O at 15 Hz). Parathion (hatched bars) inhibited vagally-induced bronchoconstriction by approximately 50% at all three frequencies (A). In contrast, paraoxon at 100 ng/kg (gray bars) and 100 µg/kg (black bars) acutely increased vagally-induced bronchoconstriction in a frequency-dependent manner (A). Paraoxon at 100 ng/kg did not potentiate ACh-induced bronchoconstriction (B) or significantly inhibit AChE activity (C). However, at 100 µg/kg, paraoxon potentiated ACh-induced bronchoconstriction (B) and inhibited AChE (C). Data are presented as mean ± SE; n = 3–4 guinea pigs (* *p*<0.05).

Intravenous administration of parathion (1 mg/kg) or paraoxon (100 ng/kg and 100 µg/kg) did not change resting inflation pressure (216. 67±27.34 mmH_2_O before and 226.67±24.30 mH_2_O after parathion; 131.4±15.0 mmH_2_0 before and 135.7±13.8 mmH_2_O after paraoxon) or heart rate (303.33±7.15 beats per min before and 303.33±8.73 beats per min after parathion; 267.1±5.9 beats per min before and 263.6±6.6 beats per min after paraoxon) in anesthetized guinea pigs. Electrical stimulation of both vagus nerves induced reproducible bronchconstrictions that were frequency dependent and atropine sensitive (data not shown), demonstrating that this effect was mediated by release of ACh onto postjunctional muscarinic receptors. Parathion inhibited vagally-induced bronchoconstriction at all frequencies tested; in contrast, paraoxon caused a dose- and frequency-dependent potentiation of vagally-induced bronchoconstriction that did not reach statistical significance (Figure 1A). Bronchoconstriction induced by intravenous acetylcholine (ACh) was reproducible, reversible and blocked by atropine (data not shown). ACh-induced bronchoconstriction was not affected by 100 ng/kg paraoxon but was potentiated by 100 µg/kg paraoxon (Figure 1B). However, this higher dose of paraoxon significantly inhibited AChE activity in circulating blood, as did parathion (Figure 1C).

**Figure 2:**
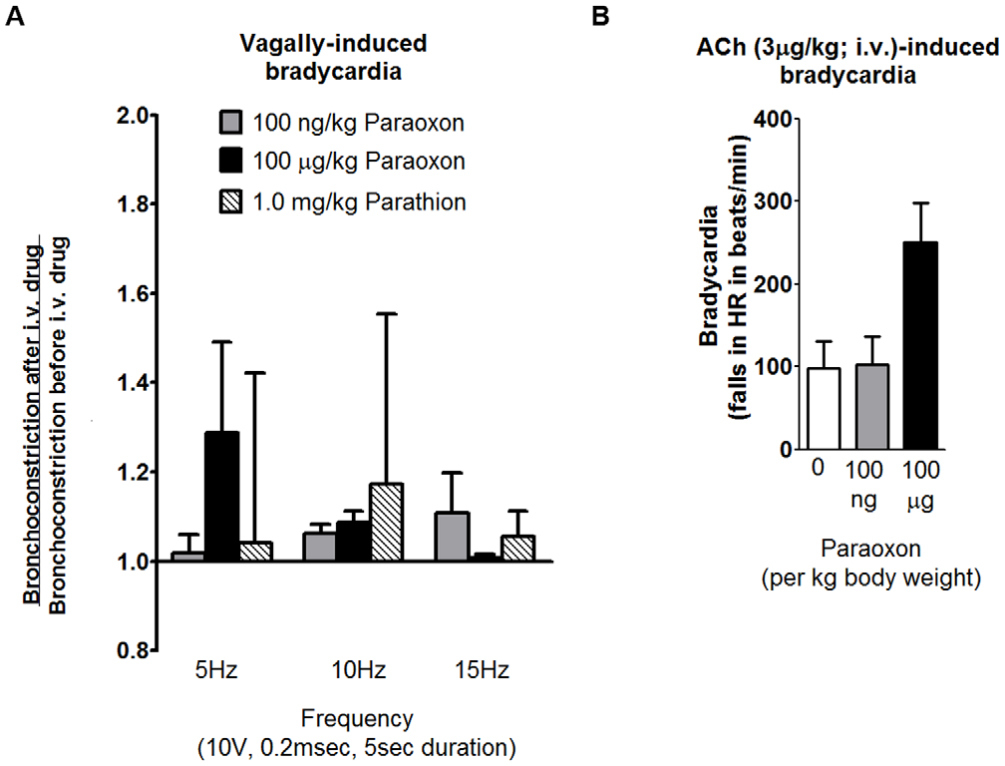
Acute intravenous administration of parathion or paraoxon did not significantly potentiate bradycardia in guinea pigs. Bradycardia was measured in anesthetized guinea pigs in response to electrical stimulation of both vagus nerves or to intravenous ACh before and after intravenous administration of parathion (1 mg/kg) or paraoxon (100 ng/kg or 100 µg/kg). None of the OP treatments had any effect on vagally-induced bradycardia at any of the frequencies tested (A). Baseline bradycardia at 5, 10, and 15 Hz was 37.62±4.13, 103.89±33.15, and 155.95±30.16 beats per minute before 100 ng/kg paraoxon administration, 47.78±22.82, 132.94±12.43, and 218.33±9.62 beats per minute before 100 µg/kg paraoxon administration, and 41.67±4.41, 85.00±7.64, and 203.33±6.67 beats per minute before 1.0 mg/kg parathion administration. (B) The higher dose of paraoxon (100 µg/kg) slightly but not significantly potentiated ACh-induced bradycardia. Data are presented as mean ± SE; n = 4–7 guinea pigs.

Electrical stimulation of vagus nerves and intravenous ACh both caused bradycardia that was reproducible and blocked by atropine (data not shown). However, no dose of parathion or paraoxon significantly altered vagally-induced bradycardia (Figure 2A). Paraoxon at 100 µg/kg but not 100 ng/kg increased ACh-induced bradycardia but this effect did not reach statistical significance (Figure 2B).

### Effects of paraoxon on activity-induced contraction in isolated trachea and ileum

**Figure 3:**
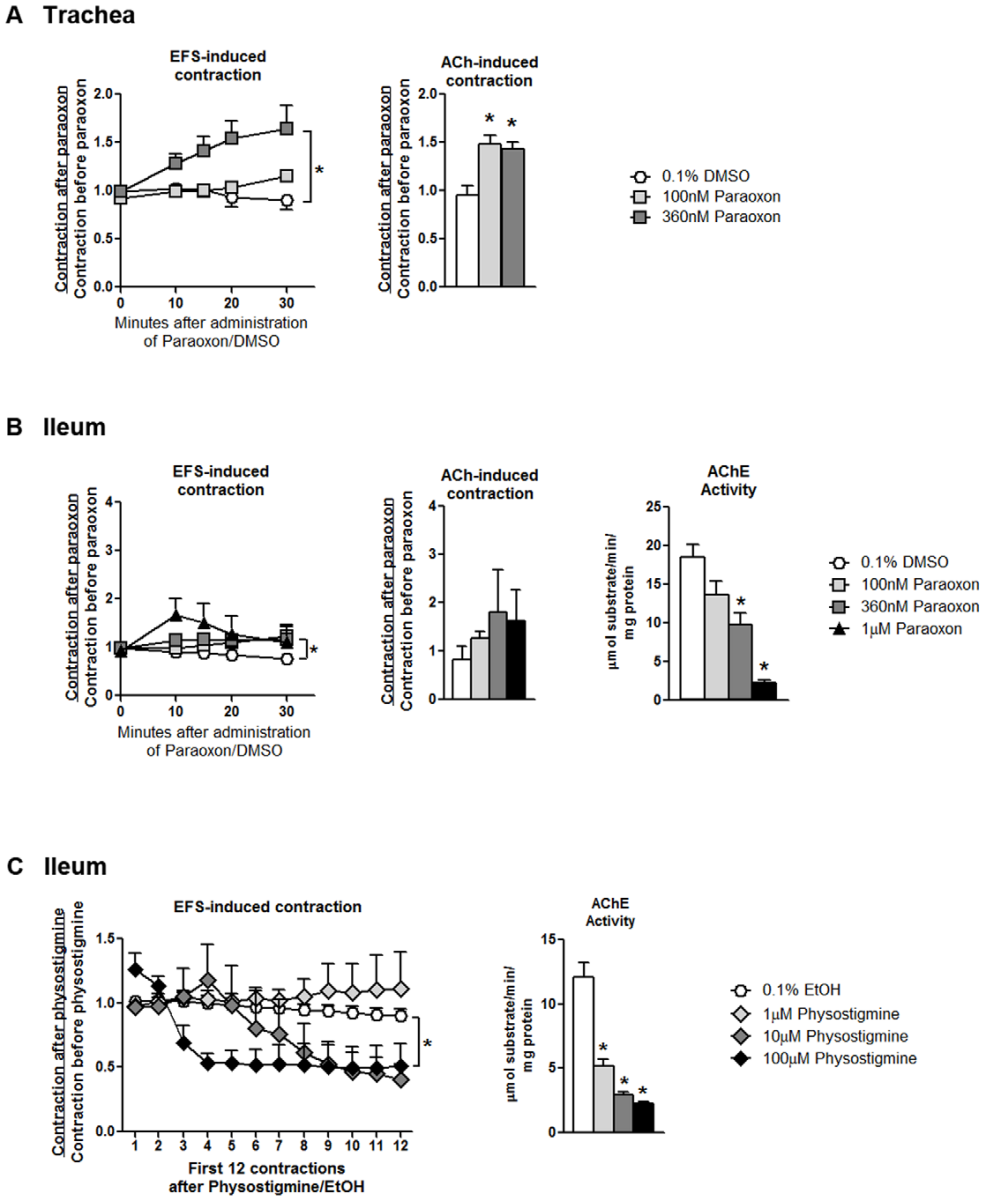
Paraoxon potentiates electrical field stimulation (EFS)-induced contractions in isolated guinea pig trachea and ileum. Contractions in response to EFS (10 Hz, 100 V, 0.2 ms, 5 s duration every 30 s) and ACh (5 µM) were measured in trachea (A) and ileum (B) before and after addition of paraoxon or vehicle (DMSO, 0.1% final). EFS induced contraction was measured for 30 min after drug treatment, while ACh-induced contractions were measured 35 min after drug treatment. (A) Paraoxon potentiated EFS-induced contractions in the trachea with significant effects at 360 nM; paraoxon at 100 and 360 nM significantly potentiated ACh-induced contractions. (B) In contrast, paraoxon did not significantly potentiate either EFS-induced or ACh-induced contractions in the ileum even at concentrations that significantly inhibited AChE activity. (C) Concentrations of physostigmine that significantly inhibited AChE activity to the same degree as paraoxon did not potentiate EFS-induced contractions in the ileum. Data are presented as mean ± SE; n = 4–6 guinea pigs (*p<0.05).

Electrical field stimulation (EFS) of isolated guinea pig trachea and ileum caused reproducible contractions that were blocked by atropine (data not shown). Exogenous ACh (5 µM) also induced atropine-sensitive contractions. Paraoxon at 360 nM but not 100 nM caused a significant, time-dependent increase in EFS-induced contractions in the trachea (Figure 3A, left panel). Paraoxon at 1.0 µM caused a less robust but statistically significant increase in EFS-induced contractions in the ileum (Figure 3B, left panel). Paraoxon at 100 and 360 nM significantly increased ACh-induced contractions in the trachea (Figure 3A, right panel); in contrast, paraoxon had no effect on ACh-induced contractions in the ileum at any concentration (Figure 3B, middle panel). Vehicle (DMSO) did not alter EFS- or ACh induced contractions in the ileum or trachea. Tissue AChE levels were measured to determine whether paraoxon-induced potentiation of contractions were related to AChE inhibition. In the ileum, paraoxon at 360 nM and 1 µM significantly inhibited AChE activity (Figure 3B, right panel). To measure detectable AChE activity in isolated trachea segments, tracheas from 2 experiments were pooled. Tracheas exposed to 0.1% DMSO, 100 nM paraoxon, and 360 nM paraoxon respectively converted on average 1.65, 0.95, and 0.6 µmol substrate/min/mg protein, suggesting paraoxon inhibited AChE activity in the trachea.

The lack of direct correlation between OP-induced potentiation of activity-induced smooth muscle contractions and inhibition of AChE activity suggested that the mechanism of increased contraction in response to OPs is not AChE inhibition. To further test this hypothesis, the effects of physostigmine, a reversible AChE inhibitor, were measured in isolated ileum. Physostigmine did not increase EFS-induced contractions (Figure 3C, left panel) at concentrations that significantly inhibited AChE activity (Figure 3C, right panel).

### Paraoxon does not modulate M2 muscarinic receptor mRNA or protein

**Figure 4:**
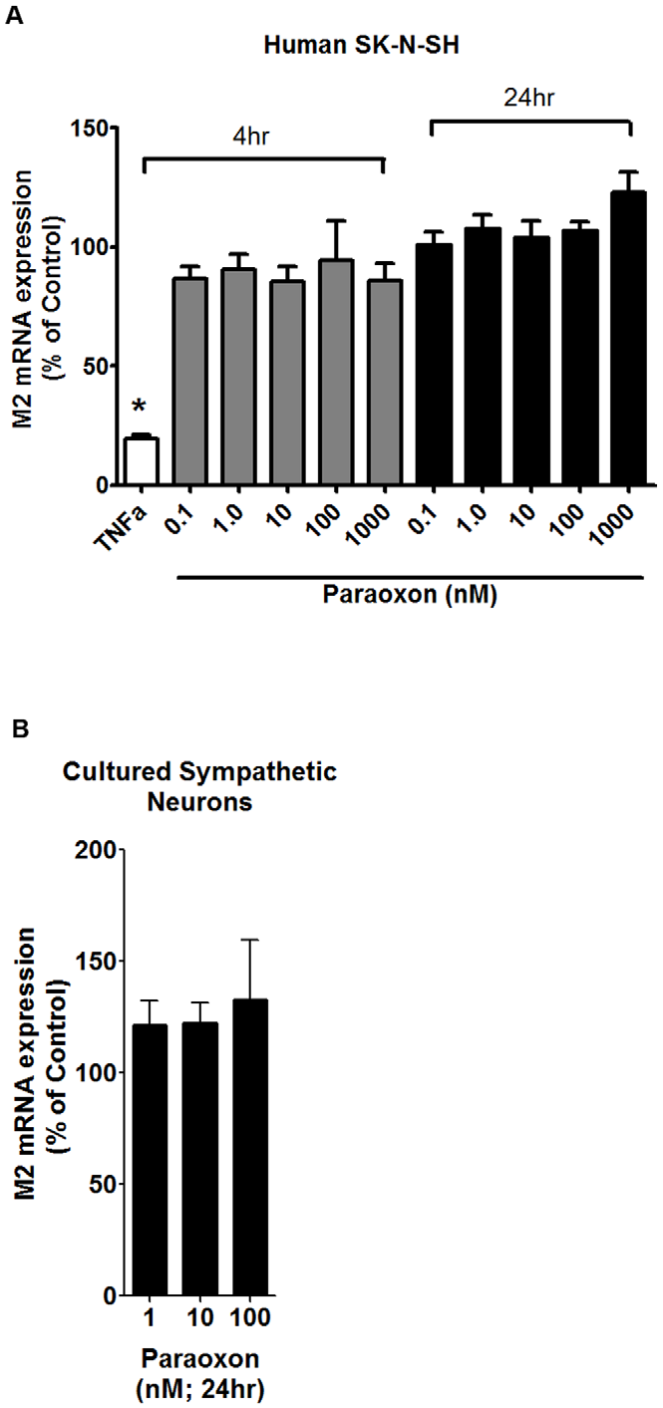
Neuronal M2 muscarinic receptor mRNA expression is not altered by paraoxon. (A) M2 receptor transcript levels in human SN-N-SH cells were not changed by exposure to paraoxon (0.1–1000 nM) for either 4 hr (gray bars) or 24 hr (black bars), while TNFα (2 ng/ml, 4 hour exposure, white bar) significantly suppressed M2 expression. (B) Exposure for 24 hr to a similar range of paraoxon concentrations had no effect on M2 mRNA levels in rat sympathetic neurons. Data are expressed as a % of control levels (cultures exposed to 0.1% DMSO) and are presented as mean ± SE; n = 3 independent cultures per treatment group (*p<0.05).

It is difficult to obtain parasympathetic neurons of sufficient homogeneity for quantitative RT-PCR analyses of neuronal mRNA [50]. Thus, to determine whether OPs might alter M2 receptor expression, M2 transcript levels were quantified by quantitative RT-PCR in SK-N-SH cells (a human neuroblastoma cell line) and in sympathetic neurons cultured from perinatal rat superior cervical ganglia (treated with anti-mitotic to remove non-neuronal cells prior to experimentation) following exposure to paraoxon (0.1–1000 nM) or vehicle (0.1% DMSO) for 4 or 24 hr. Paraoxon had no significant effect on expression of mRNA encoding M2 receptors in either SK-N-SH cells (Figure 4A) or sympathetic neurons (Figure 4B). In contrast, as previously reported [51], M2 mRNA levels were significantly decreased in SK-N-SH cells following a 4 hr exposure to TNFα (2 ng/ml).

**Figure 5:**
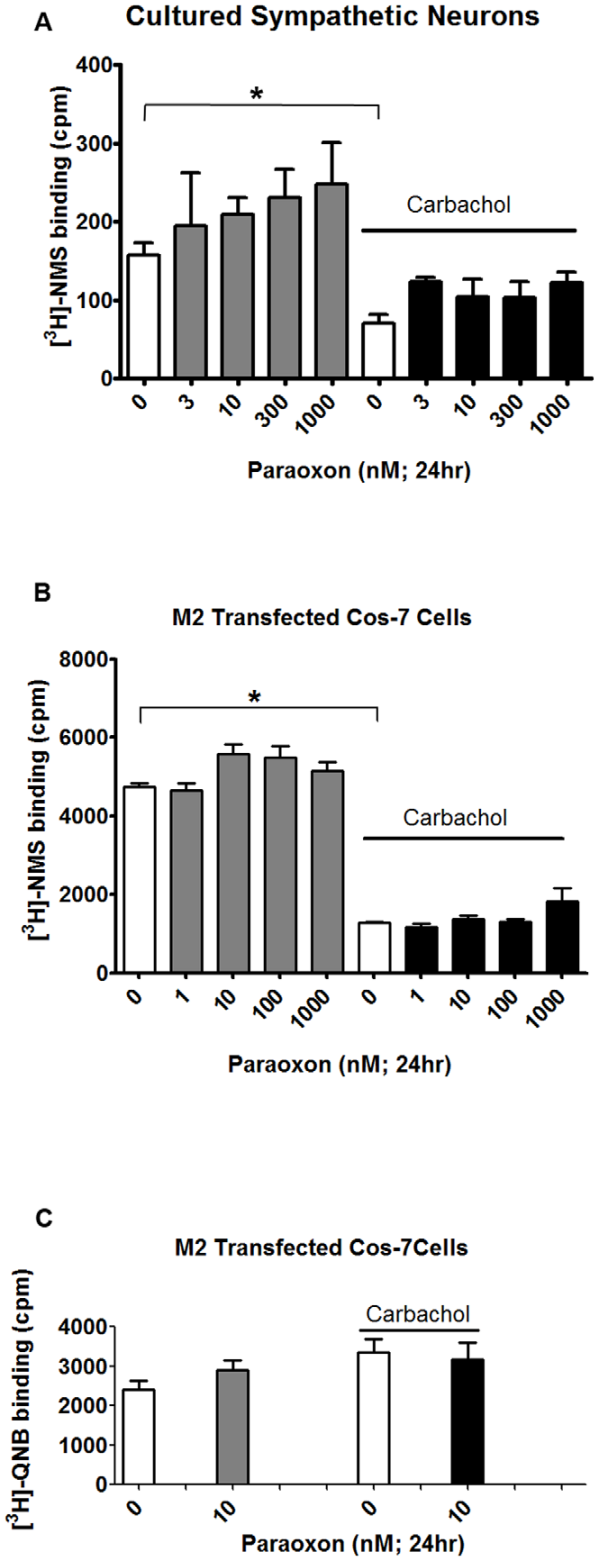
Paraoxon does not alter expression of M2 muscarinic receptor protein. Rat sympathetic neurons in cell culture (A) or Cos-7 cells transfected with full length cDNA encoding human M2 receptor (B) were treated with paraoxon (1–1000 nM) or vehicle (0.1% DMSO) for 24 hr in the absence or presence of carbachol (1 mM). Muscarinic receptor expression was determined as specific binding of [^3^H]-NMS (1 nM; surface receptors) or [^3^H]-QNB (1 nM; total receptors) in the absence (total) or presence (non-specific binding) of atropine (0.1 M). In both cell types, carbachol significantly decreased [^3^H]-NMS (A and B, open bars), but not [^3^H]-QNB (C, open bars) binding. In both sympathetic neurons (A) and Cos-7 cells (B), paraoxon had no effect on [^3^H]-NMS or [^3^H]-QNB binding in the absence or presence of carbachol. Data are represented as mean ± SE; n = 3–5.

Muscarinic receptor expression was also measured at the protein level in primary cultures of purified sympathetic neurons (Figure 5A) and in Cos-7 cells expressing cDNA encoding the full length human M2 receptor sequence (Figure 5 B and C). Cultures were exposed to paraoxon or vehicle (0.1% DMSO) for 24 hr in the absence or presence of the muscarinic agonist carbachol (1 mM). Carbachol alone significantly decreased [^3^H]-NMS binding in both sympathetic neurons and Cos-7 cells (Figure 5A and B, white bars) but did not affect [^3^H]-QNB binding in the Cos-7 cells (Figure 5C, white bars). Paraoxon alone did not affect [^3^H]-NMS in either sympathetic neurons or Cos-7 cells (Figure 5A and B, gray bars) or [^3^H]-QNB binding in Cos-7 cells (Figure 5C, gray bars). Neither did paraoxon affect carbachol-induced decreases in [^3^H]-NMS binding in either cell type (Figure 5A and B, black bars).

### M2 muscarinic receptors are not phosphorylated

**Figure 6:**
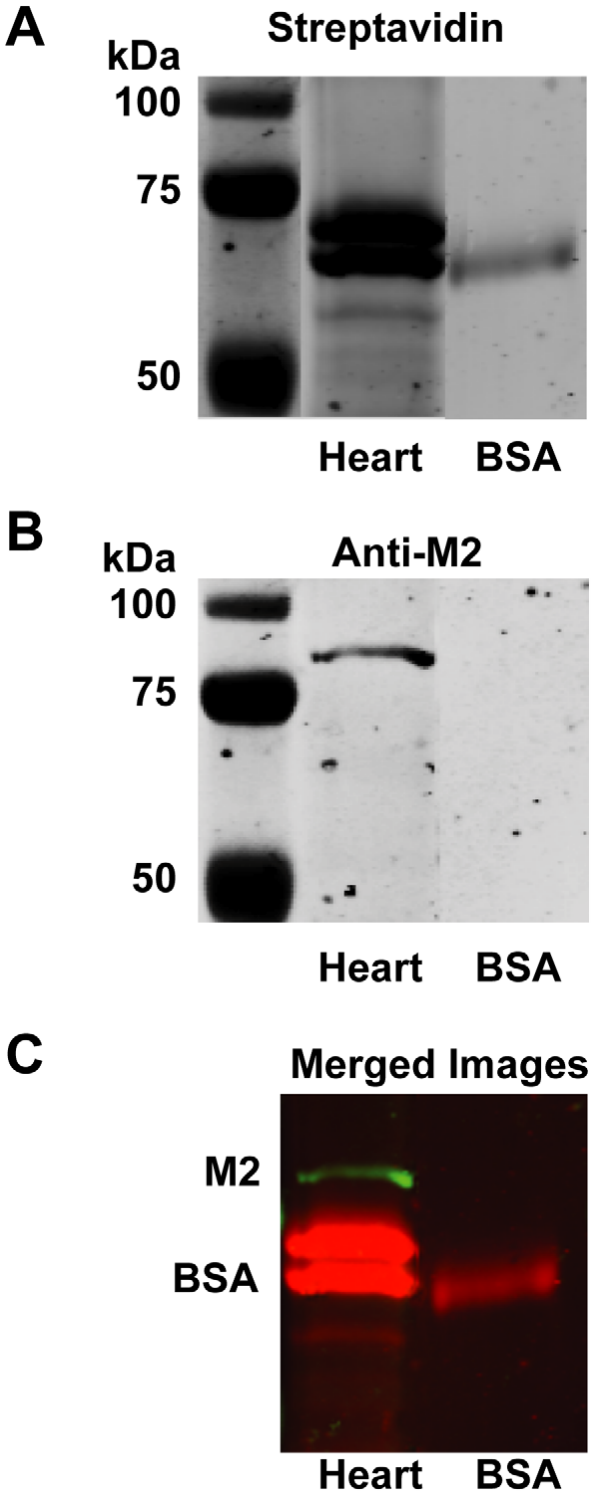
The organophosphorus fluorophosphonate (FP) probe does not phosphorylate M2 muscarinic receptors in guinea pig heart. Guinea pig heart cell membrane preparations with abundant expression of M2 muscarinic receptors or BSA were reacted with a fluorophosphonate tethered to a biotin group (FP-biotin) for 24 hr prior to separation by acrylamide gel electrophoresis. Blots of these gels were probed with streptavidin tagged with an infrared fluorophore (A, red in the merged image in panel C) to localize the biotin tag and with anti-M2 receptor antibody conjugated to a different infrared fluorophore (B, green in the merged image in panel C). As indicated in the merged image (C), proteins in the heart membranes that were biotinylated by the FP probe (red) did not co-localize with bands recognized by the anti-M2 receptor antibodies (green).

Previous studies have shown that a biotinylated organophosphorus fluorophosphonate (FP-biotin) reacts with AChE, phosphorylating the critical serine residue that is targeted by OPs to inhibit the enzymatic activity of AChE [52], and that it can be used as a probe for identifying other proteins that are phosphorylated by OPs, including bovine serum albumin (BSA) [53]. Therefore, to test the hypothesis that parathion or paraoxon inhibit M2 receptor function via phosphorylation, guinea pig heart membranes were reacted with FP-biotin. BSA (0.5 mg/ml) was also incubated with the FP-biotin as a positive control. Protein blots were reacted with streptavidin tagged with an infrared fluorophore to visualize bands containing biotinylated proteins (Figure 6; top panel) and re-probed with anti-M2 receptor antibody to identify bands corresponding to the M2 receptor (Figure 6; middle panel). Streptavidin reacted with BSA and with several proteins in heart membranes, demonstrating that FP-biotin phosphorylated proteins in the heart membrane. Antibody specific for the M2 receptor did not bind to BSA, but did bind to a protein in heart membranes with a molecular weight consistent with that of the M2 receptor (Figure 6, middle panel). However, there was no overlap between proteins in the heart membranes that reacted with the FP-biotin and those recognized by the M2 receptor antibody (Figure 6, bottom panel).

## Discussion

We have previously established that the organophosphorus pesticides chlorpyrifos, diazinon and parathion cause airway hyperreactivity in guinea pigs [26], [27]. This effect is mediated by blockade of inhibitory M2 muscarinic receptors [26] that normally function to inhibit acetylcholine release from airway parasympathetic nerves [34]. A significant body of literature shows that OPs can interact directly with muscarinic receptors in the brain and heart, although the nature of this interaction (agonism, competitive antagonism or allosteric inhibition) varies according to OP and cell type (reviewed in [46], [54]). Thus, our initial hypothesis was that OPs interact directly with M2 receptors on parasympathetic nerves in the airways to antagonize M2 receptor function or downregulate M2 receptor expression.

To test for direct antagonism, we first determined whether parathion or its oxon metabolite, paraoxon, caused acute potentiation of nerve-induced airway smooth muscle contractions. In contrast to our previous demonstration that parathion potentiates vagally-induced bronchoconstriction in guinea pigs 24 hr after a single subcutaneous injection at 1 mg/kg [26], [44], acute i.v. administration of parathion at the same dose inhibited bronchoconstriction by 50% (Figure 1). The mechanism(s) underlying the paradoxical effect of acutely administered parathion is unlikely to include blockade of nerve conduction given that vagally-induced bradycardia was not similarly decreased (Figure 2). While the lack of effect on vagally-induced bradycardia suggests that parathion is not blocking postjunctional M2 receptors in the heart, acute i.v. parathion may be antagonizing postjunctional M3 receptors [55]–[58] or inhibiting signaling cascades that mediate airway smooth contraction [55], [58]–[60]; however, the exact mechanism has yet to be determined.

In contrast, acute i.v. administration of paraoxon increased vagally-induced bronchoconstriction in a frequency and dose-dependent manner (Figure 1). The increase was consistent but small and not statistically significant. It is unlikely that this potentiation was due to inhibition of AChE since paraoxon had no (100 ng/ml) or a small (100 µg/ml) effect on AChE activity in the blood. In addition, it has been shown that inhibition of AChE actually decreases acetylcholine release from airway nerves due to increased activity of the inhibitory neuronal M2 receptors [61]. While these data support the possibility that these OPs interfere directly with neuronal M2 receptor function in airways, they do not explain the robust effects observed 24 hr and 7 d after subcutaneous OP administration [26], [27].

To further explore the hypothesis that OPs pharmacologically antagonize M2 receptors in autonomic nerves, we examined the direct effects of paraoxon on smooth muscle contraction in isolated trachea. These organ bath experiments allow the direct application of OPs to tracheal muscle, avoiding any dilution effects with i.v. administrations. Paraoxon increased EFS-induced tracheal muscle contraction, but this effect was slow in onset and the same treatment also potentiated ACh-induced muscle contraction (Figure 3). Because of the low levels of AChE activity in the trachea, we were unable to reliably measure AChE activity, thus we cannot exclude the possibility that paraoxon was potentiating tracheal smooth muscle contraction via AChE inhibition. Therefore, we repeated these studies in isolated ileum, which also express inhibitory M2 receptors on parasympathetic nerves that regulate smooth muscle contraction [62]. Paraoxon at 360 nM but not at 100 nM or 1 µM increased EFS-induced ileal smooth muscle contraction; while this increase was statistically significant, it was of such small magnitude that it is unlikely to be physiologically relevant (Figure 3). No concentration of paraoxon increased ACh-induced contraction despite significant inhibition of AChE at the two higher doses. Similarly, concentrations of the AChE inhibitor physostigmine that significantly inhibited AChE had no potentiating effect on EFS-induced ileal smooth muscle contraction (Figure 3C). Collectively, these *in vivo* and *ex vivo* data indicate there is no relationship between AChE inhibition and potentiation of nerve-induced smooth muscle contraction in the lung, heart or ileum, which is consistent with our previous findings [26], [27]. Moreover, these data do not support the hypothesis that OPs directly antagonize M2 function in autonomic nerves.

Muscarinic receptor function is dependent on receptor expression that can be regulated at both transcriptional [51] and post-translational levels [63], [64]; however, our data do not support any of these mechanisms for OP-induced M2 receptor dysfunction in airways observed 24 hr or 7d post-exposure [26], [27]. Specifically, paraoxon did not change transcript levels for M2 receptors in the human SK-N-SH neuronal cell line or in primary rat sympathetic nerve cultures (Figure 4). Although carbachol induced internalization of M2 receptors in sympathetic nerves and Cos-7 cells expressing human M2 receptors, paraoxon did not cause M2 receptor internalization or interfere with carbachol-induced receptor internalization (Figure 5), which is consistent with previous reports that chlorpyrifos oxon also did not alter receptor internalization in several different cell lines transfected with cDNA encoding M2 receptors [63]. In contrast to previous reports suggesting that chlorpyrifos directly phosphorylates M2 receptors in rat heart [64], we did not observe phosphorylation of guinea pig cardiac M2 receptors using an FP-biotin probe [52]. The lack of covalent modification of M2 receptors may be due to a number of factors, including less access to reactive M2 residues by the large and lipophilic FP-biotin or a change in leaving group from trichloropyridinol (chlorpyrifos) to fluoride (FP-biotin). It is also possible that there is no discrepancy between the previous study and our findings since the phosphorylated band pulled down from lysates of rat heart treated *in vitro* with chlorpyrifos [64] was not re-probed with M2 specific antibodies to confirm its identity as the M2 receptor.

Although the current paradigm is that OPs interact directly with muscarinic receptors to either stimulate or inhibit their function (reviewed in [46], [54]), we are unable to demonstrate any direct interaction between the active metabolite of parathion and M2 muscarinic receptors. This adds to the growing body of literature suggesting that regulation of M2 receptors [65]–[68]and effects of OPs on muscarinic receptors [57], [63], [69]–[71] vary with tissue and cell type. None-theless, when measured 24 hr or 7 d post-exposure, OPs cause airway hyperreactivity *in vivo* and this effect is mediated by loss of M2 receptor function in parasympathetic nerves innervating airway smooth muscle [26], [27]. The time course suggests that the effects of OPs on neuronal M2 receptors require intermediate cells, such as inflammatory cells. We have shown in guinea pigs that are sensitized to ovalbumin that eosinophils mediate OP-induced airway hyperreactivity; however, eosinophils are not necessary for OP-induced airway hyperreactivity in non-sensitized guinea pigs [44]. There are other inflammatory cell types resident in the lungs including macrophages, mast cells and neutrophils, all of which have been implicated in airway hyperreactivity triggered by other stimuli [72], including viral infection [73] and ozone exposure [74].

There is experimental evidence that OPs can affect inflammatory cells. Malathion stimulates mouse macrophages to generate reactive oxygen species (ROS) and cathepsin D [75]–[77], and potentiates macrophage phagocytosis [78]. Malathion also stimulates mast cell degranulation in the intestine and skin [79] and histamine release from mast cells [80] and basophils [81]. *In vivo*, OP activation of macrophages may occur indirectly via mast cells or prostaglandins, since depletion of the former or blockade of the latter prevents malathion-induced macrophage activation in mice [82]. Inhalation of sarin, an OP nerve agent, induces an inflammatory response in the lungs of guinea pigs [83] and rats[84], measured as increased levels of inflammatory mediators, such as histamine and prostaglandins; increased numbers of inflammatory cells, such as eosinophils and macrophages; or increased mRNA expression for pro-inflammatory cytokines, such as IL-1β, IL-6 and TNFα. Studies of neutrophil activity from OP-exposed workers suggest that neutrophil chemotaxis may be decreased [85], although *ex vivo* exposure of human whole blood cultures, containing neutrophils and other inflammatory cells, to chlorpyrifos potentiated LPS-induced release of IFNγ [86]. Collectively, these observations strongly suggest that OPs may increase inflammatory cytokines in the airways via stimulation of inflammatory cells. Since it is known that proinflammatory cytokines such as IFNγ, TNFα and IL-1β can decrease M2 expression and function *in vivo* [47], [51], [87], we hypothesize that these other inflammatory cells may be critical to OP-induced airway hyperreactivity in non-sensitized individuals. Future experiments should address whether these multiple, inflammatory pathways mediate the effects of OPs on neuronal M2 receptor dysfunction in airways.

## Materials and Methods

### Animals

All protocols involving animals were approved by the Animal Care and Use Committees at Oregon Health & Science University and the University of California at Davis. Specific pathogen-free female Dunkin-Hartley guinea pigs (300–350 g) were purchased from Elm Hill Labs (Chelmsford, MA) and housed in rooms with high-efficiency particulate-filtered air. Timed-pregnant Sprague-Dawley rats were purchased from Charles River (Wilmington, MA) and maintained under standard housing conditions with *ad libitum* access to food and water.

### OPs

Parathion (*o*,*o*-diethyl-*o*-*p*-nitrophenyl phosphorothioate, 99.5% pure) and paraoxon (diethyl-p-nitrophenylphosphate, 98.6% pure) were purchased from Chem Service (West Chester, PA) and used prior to the expiration date with interim storage as recommended by the manufacturer. These OPs were suspended in dimethyl sulfoxide (DMSO) and diluted in buffer or tissue culture media immediately prior to experiments such that final DMSO concentrations never exceeded 0.1%. The biotinylated organophosphorus fluorophosphonate, FP-biotin, [10-(fluoroethoxyphosphinyl)-N-(biotinamidopentyl)decanamide] was synthesized as previously described [52].

### *In vivo* measurement of bronchoconstriction and bradycardia

Physiological experiments measuring airway function in guinea pigs were performed as previously described [88]. Guinea pigs were chosen for these studies because their lung pharmacology is similar to humans [89]. In addition, organophosphorus pesticides uniformly cause airway hyperreactivity in guinea pigs unlike mice which respond differently dependent upon the strain [90], [91]. Guinea pigs were anesthetized with urethane (1.9 g/kg ip, Sigma-Aldrich, St. Louis, MO), tracheostomized, and mechanically ventilated with positive pressure and constant volume (100 breaths/min; 1 ml volume/100 g body weight). Jugular veins were cannulated for drug administration, and a carotid artery was cannulated to monitor blood pressure and heart rate. Both vagus nerves were cut and the distal ends placed on electrodes and submerged in mineral oil. Pulmonary inflation pressure was measured using a pressure transducer (Becton Dickinson, Franklin Lakes, NJ) attached to a side arm of the tracheal cannula. Bronchoconstriction was measured as an increase in pulmonary inflation pressure (in mm H_2_O) over baseline ventilator pressure.

The vagus nerves were stimulated at one minute intervals (10 V, 10 Hz, 0.2 ms, 5 s duration) for a maximum of 10 min until vagally-induced bronchoconstriction became consistent. After this initial warm up, bronchoconstriction and bradycardia in response to electrical stimulation of the vagus nerves (3 times each at 5, 10 and 15 Hz; at 10V, 0.2 ms, 5 s duration) and then to 2 administrations of exogenous ACh (3 µg/kg, i.v.) was measured. This sequence of vagally-induced bronchoconstriction followed by ACh-induced bronchoconstriction was repeated until two reproducible sequences were recorded, and then either paraoxon (100 ng/kg or 100 µg/kg) or parathion (1 mg/kg) was administered intravenously. The doses of paraoxon were calculated based on our previous findings that 10 µg/kg parathion caused airway hyperreactivity 24 hr after exposure [44], and that paraoxon is 100–1000 times more potent than parathion in inhibiting AChE activity [92]. Within 1 min after OP administration, another sequence of vagally- and ACh-induced bronchoconstriction was recorded and bronchoconstric-tion at each frequency and ACh administration were averaged.

### Organ bath experiments

Experiments with isolated tissues were carried out as previously described [93]. Briefly, guinea pigs were killed by overdose of sodium pentobarbital (150 mg/kg; i.p.). The trachea and ileum were removed and cut transversely into sections consisting of 3–5 cartilaginous rings (trachea) or segments measuring 1–1.5 cm (ileum). Tissues were mounted horizontally (trachea) or vertically (ileum) between zigzag platinum electrodes and attached to a high sensitivity force displacement transducer (Radnotti Glass Technology, Inc., Monrovia, CA) in organ baths containing 5 ml Krebs-Henseleit solution and allowed to equilibrate for 30 min under 1.0 g tension.

Contractions were measured as an increase in tension from baseline. The reference for ACh-induced contraction is the mean of three peak contractions in response to 5 µM ACh (Acros Organics, Fair Lawn, NJ) at the start of each experiment. The reference for EFS-induced contractions were the mean of 5 repetitive stimulations of ileum (10 Hz, 100 V, 0.2 ms, 5 s duration every 30 s) and trachea (10 Hz, 100 V, 0.2 ms, 15 s duration every 60 s), immediately following a 10–15 min period of repetitive stimulation to allow tissues to achieve consistent contractions.

Paraoxon (0.1–1 µM, final concentration), DMSO (0.1%, final concentration), physostigmine (1–100 µM final concentrations; Sigma-Aldrich) or 0.1% ethanol (vehicle for physostigmine) were added to the baths, and 3 consecutive EFS-induced contractions were recorded 0, 10, 15, 20, and 30 min after drug application. At the end of the experiment, the stimulator was turned off and 5 µM ACh-induced contractions were measured. Trachea and ileum contractions induced by EFS and ACh were compared before (baseline) and after paraoxon, DMSO, physostigmine or ethanol. Blockade of EFS-induced and ACh-induced contractions by 100 µM atropine (Sigma-Aldrich) confirmed that all responses were mediated via muscarinic receptor activation.

### Acetylcholinesterase (AChE) activity

Following physiological experiments, lungs were perfused with PBS, dissected, frozen on dry ice and stored at −80°C. At the end of the organ bath experiments, trachea and ileum segments were blotted on filter paper, frozen on dry ice and stored at −80°C. On the day of analysis, samples were thawed on ice, and AChE activity measured using the Ellman assay [94] as previously described [26].

### Tissue Culture

The human neuroblastoma SK-N-SH cells (ATCC, Manassas, VA) were maintained in Minimum Essential Medium containing 10% fetal bovine serum (FBS), nonessential amino acids, 1 mM sodium pyruvate, 100 U/ml penicillin, 100 µg/ml streptomycin, and 250 ng/ml amphotericin B (Mediatech, Inc., Manassas, VA). Sympathetic neurons were dissociated from superior cervical ganglia of perinatal rats and maintained in serum-free media containing 125 ng/ml nerve growth factor as previously described [95]. Cos-7 cells (ATCC, Manassas, VA) were conditioned to grow in serum free media (VP-SFM, Invitrogen) and were transfected with pCDNA encoding the full-length human M2 muscarinic receptor cDNA [96] using Fugene6 transfection reagent (Roche, Nutley, NJ) as described by the manufacturer. Transfection efficiency was routinely 70–80%.

### Quantitative reverse transcriptase polymerase chain reaction (RT-PCR)

SK-N-SH cells were exposed to 0.1–1000 nM paraoxon, 0.1% DMSO, or 2 ng/ml human tumor necrosis factor α (TNFα; Sigma-Aldrich) for 4 or 24 hr at 37°C in 95% O_2_/5% CO_2_. Rat sympathetic neurons (7 days *in vitro*) were exposed to 1–100 nM paraoxon or 0.1% DMSO for 24 hr at 35°C at 95% O_2_/5% CO_2_. RNA was isolated and reverse transcribed as previously described [51]. cDNA (2 µl of a 1∶10 dilution for M2 muscarinic receptor and 1 µl of a 1∶100 dilution for 18S) was amplified for 45 cycles at 58°C using QuantiTect SYBR Green PCR kit (Qiagen, Valencia, CA) in duplicate using the 7500 Fast Real-Time PCR System (Applied Biosystems, Foster City, CA). PCR products were quantified using the Mx3000P real-time PCR System (Stratagene, La Jolla, CA). Specific real-time PCR primers for the M2 muscarinic receptor and 18S rRNA were synthesized (Integrated DNA Technologies, Coralville, IA) as follows: human M2 5′ CAAAGGTCACACACCACAGG and human M2 3′ TTAAAGTCAACCGCCACCTC; rat M2 5′TACCCAGTTAAGCGGACCAC and rat M2 3′GCAGATAGAACGCTGCAATG; 18S rRNA 5′ GTAACCCGTTGAACCCCATT and 18S rRNA 3′ CCATCCAATCGGTAGTAGCG. The relative amount of RNA was calculated from the slope of a standard curve for each product and normalized to individual 18S RNA expression.

### Muscarinic receptor expression assays

Surface expression of muscarinic receptors was determined using tritiated scopolamine methyl chloride ([^3^H]-NMS; Perkin Elmer, Waltham, MA) whereas total muscarinic receptor expression was determined using tritiated quinuclidinyl benzilate ([^3^H]-QNB; Perkin Elmer) as previously described [97]. Both rat sympathetic neurons and Cos-7 cells were treated with either paraoxon (1–1000 nM) or vehicle alone (0.1% DMSO) for 24 hr at 37°C in the presence or absence of the muscarinic agonist carbachol (1 mM, Calbiochem, San Diego, CA). Cells were subsequently reacted with 1 nM [^3^H]-NMS for 4 hr at 4°C or with [^3^H]-QNB for 1 hr at 37°C in the absence or presence of atropine (0.1 M, Sigma). The cells were lysed with 1% Triton X-100 and radioactivity in cell lysates was quantified. Data are presented as the specific binding, which was determined as counts per minute (cpm) in the absence of atropine minus cpm in the presence of atropine normalized to cell count (number of cells plated in each culture). Every experiment was repeated 3–5 times with 4–6 individual cultures per experiment.

### Fluorophosphonate-biotin labeling in heart tissue

Guinea pig heart membranes were used for these experiments because they express a high density of M2 receptors [98]. Hearts were perfused with PBS, minced, suspended in 5 mM 4-(2-hydroxyethyl)-1-piperazineethanesulfonic acid (HEPES; pH 7.4) and homogenized on ice. Homogenates were centrifuged at 10,000×g for 10 min at 4°C, and supernatants further centrifuged at 40,000×g for 30 min at 4°C. Pelleted heart membranes (1 mg total protein) were resuspended in 500 µl of 20 mM Tris (pH 7.4). Heart membranes and bovine serum albumin (BSA; 0.5 mg/ml) were reacted with FP-biotin (20 µM) for 24 hr at 20–25°C in the dark. FP-biotin was reacted with BSA as a positive control for detection of phosphorylation by FP-biotin [52]. Proteins were denatured with 5X sample buffer (10% SDS, 10 mM β-mercaptoethanol, 20% glycerol, 0.2 M Tris-HCl, pH 6.8) containing 8 M urea, separated on a 12% acrylamide gel containing 4 M urea, and transferred to PDVF membrane. Protein blots were blocked for 1 hr in Odyssey buffer (Licor, Lincoln, NE) and incubated overnight at 4°C with a rabbit anit-M2 muscarinic receptor antibody (M9558; Sigma-Aldrich) diluted 1∶500 in buffer containing 0.l% Tween-20. After several washes in PBS containing 0.1% Tween-20, protein blots were incubated for 2 hr at 20 –25°C with an IR700-conjugated goat anti-rabbit secondary antibody (1∶1000; Rockland Immunochemical, Gilbertsville, PA) and an IR800-conjugated strep avidin (1∶5000; Rockland) in Odyssey buffer containing 0.1% Tween-20 and 0.01% sodium dodecyl sulfate. Immunoblots were washed and imaged on an Infrared Imaging System (Licor). Images of FP-biotin and M2 muscarinic receptor labeling were overlaid using Metamorph Imaging System (Universal Imaging Corp., Downingtown, PA).

### Data analysis

EFS-induced ileum and trachea contractions were analyzed by two-way analysis of variance (ANOVA) with repeated measures. Vagally- and ACh-induced broncho-constrictions were analyzed by Student's T-test. All other data were analyzed by one-way ANOVA using the Bonferroni correction post-hoc test. Statistical probability of p<0.05 was considered significant.

## References

[1] Hartert TV, Peebles RS (2000). Epidemiology of asthma: the year in review.. Curr Opin Pulm Med.

[2] Weitzman M, Gortmaker SL, Sobol AM, Perrin JM (1992). Recent trends in the prevalence and severity of childhood asthma.. Jama.

[3] Koch D, Lu C, Fisker-Andersen J, Jolley L, Fenske RA (2002). Temporal association of children's pesticide exposure and agricultural spraying: report of a longitudinal biological monitoring study.. Environ Health Perspect.

[4] Fenske RA, Lu C, Barr D, Needham L (2002). Children's exposure to chlorpyrifos and parathion in an agricultural community in central Washington State.. Environ Health Perspect.

[5] Wilhoit L, Davidson N, Supkoff D, Steggal J, Braun A (1999). Pesticide Use Analysis and Trends from 1991 to 1996. Sacramento, CA: State of California Environmental Protection Agency.. PM 99-01 PM.

[6] USDA (2003). Agricultural chemicals and production technology.. Economic Research Service.

[7] Weisenburger DD (1993). Human health effects of agrichemical use.. Hum Pathol.

[8] Whyatt RM, Camann DE, Kinney PL, Reyes A, Ramirez J (2002). Residential pesticide use during pregnancy among a cohort of urban minority women.. Environ Health Perspect.

[9] Berkowitz GS, Obel J, Deych E, Lapinski R, Godbold J (2003). Exposure to Indoor Pesticides during Pregnancy in a Multiethnic, Urban Cohort.. Environ Health Perspect.

[10] Lu C, Knutson DE, Fisker-Andersen J, Fenske RA (2001). Biological monitoring survey of organophosphorus pesticide exposure among pre-school children in the Seattle metropolitan area.. Environ Health Perspect.

[11] CDC (2003). Second National Report on Human Exposure to Environmental Chemicals. National Center for Environmental Health.. NCEH 03-0022 NCEH.

[12] National Research Council CoACDTC (1993). Alternative Technologies for the Destruction of Chemical Agents and Munitions; Board on Army Science and Technology, Commission on Engineering and Technical Systems..

[13] Arruda LK, Vailes LD, Ferriani VP, Santos AB, Pomes A (2001). Cockroach allergens and asthma.. J Allergy Clin Immunol.

[14] Eggleston PA, Arruda LK (2001). Ecology and elimination of cockroaches and allergens in the home.. J Allergy Clin Immunol.

[15] Bryant DH (1985). Asthma due to insecticide sensitivity.. Aust N Z J Med.

[16] Deschamps D, Questel F, Baud FJ, Gervais P, Dally S (1994). Persistent asthma after acute inhalation of organophosphate insecticide.. Lancet.

[17] Hoppin JA, Umbach DM, London SJ, Alavanja MC, Sandler DP (2002). Chemical predictors of wheeze among farmer pesticide applicators in the Agricultural Health Study.. Am J Respir Crit Care Med.

[18] Hoppin JA, Umbach DM, London SJ, Henneberger PK, Kullman GJ (2007). Pesticides and Atopic and Non-atopic Asthma Among Farm Women in the Agricultural Health Study.. Am J Respir Crit Care Med.

[19] Hoppin JA, Umbach DM, London SJ, Lynch CF, Alavanja MC (2006). Pesticides and adult respiratory outcomes in the agricultural health study.. Ann N Y Acad Sci.

[20] Hoppin JA, Umbach DM, London SJ, Lynch CF, Alavanja MC (2006). Pesticides associated with wheeze among commercial pesticide applicators in the Agricultural Health Study.. Am J Epidemiol.

[21] Hoppin JA, Valcin M, Henneberger PK, Kullman GJ, Umbach DM (2007). Pesticide use and chronic bronchitis among farmers in the agricultural health study.. Am J Ind Med.

[22] O'Malley M (1997). Clinical evaluation of pesticide exposure and poisonings.. Lancet.

[23] Salam MT, Li YF, Langholz B, Gilliland FD (2004). Early-life environmental risk factors for asthma: findings from the Children's Health Study.. Environ Health Perspect.

[24] Gustin P, Dhem AR, Lomba F, Lekeux P, Van de Woestijne KP (1988). Measurement of total respiratory impedance in calves by the forced oscillation technique.. J Appl Physiol.

[25] Segura P, Chavez J, Montano LM, Vargas MH, Delaunois A (1999). Identification of mechanisms involved in the acute airway toxicity induced by parathion.. Naunyn Schmiedebergs Arch Pharmacol.

[26] Lein PJ, Fryer AD (2005). Organophosphorus insecticides induce airway hyperreactivity by decreasing neuronal M2 muscarinic receptor function independent of acetylcholinesterase inhibition.. Toxicol Sci.

[27] Fryer AD, Lein PJ, Howard AS, Yost BL, Beckles RA (2004). Mechanisms of organophosphate insecticide-induced airway hyperreactivity.. Am J Physiol Lung Cell Mol Physiol.

[28] Ernst P (2002). Pesticide exposure and asthma.. Am J Respir Crit Care Med.

[29] Senthilselvan A, McDuffie HH, Dosman JA (1992). Association of asthma with use of pesticides. Results of a cross- sectional survey of farmers.. Am Rev Respir Dis.

[30] Casarett LJ, Doull J (1975). Toxicology..

[31] Coulson FR, Fryer AD (2003). Muscarinic acetylcholine receptors and airway diseases.. Pharmacol Ther.

[32] Roffel AF, Elzinga CR, Zaagsma J (1990). Muscarinic M3 receptors mediate contraction of human central and peripheral airway smooth muscle.. Pulm Pharmacol.

[33] Roffel AF, Meurs H, Zaagsma J (1994). Muscarinic acetylcholine receptors and control of smooth muscle tone.. Trends Pharmacol Sci.

[34] Fryer AD, Maclagan J (1984). Muscarinic inhibitory receptors in pulmonary parasympathetic nerves in the guinea-pig.. Br J Pharmacol.

[35] Minette PA, Barnes PJ (1988). Prejunctional inhibitory muscarinic receptors on cholinergic nerves in human and guinea pig airways.. J Appl Physiol.

[36] Gambone LM, Elbon CL, Fryer AD (1994). Ozone-induced loss of neuronal M2 muscarinic receptor function is prevented by cyclophosphamide.. J Appl Physiol.

[37] Fryer AD, Wills-Karp M (1991). Dysfunction of M2-muscarinic receptors in pulmonary parasympathetic nerves after antigen challenge.. J Appl Physiol.

[38] Jacoby DB, Fryer AD (1991). Virus-induced airway hyperresponsiveness–possible involvement of neural mechanisms.. Am Rev Respir Dis.

[39] Minette PA, Lammers JW, Dixon CM, McCusker MT, Barnes PJ (1989). A muscarinic agonist inhibits reflex bronchoconstriction in normal but not in asthmatic subjects.. J Appl Physiol.

[40] Costello RW, Schofield BH, Kephart GM, Gleich GJ, Jacoby DB (1997). Localization of eosinophils to airway nerves and effect on neuronal M2 muscarinic receptor function.. Am J Physiol.

[41] Evans CM, Fryer AD, Jacoby DB, Gleich GJ, Costello RW (1997). Pretreatment with antibody to eosinophil major basic protein prevents hyperresponsiveness by protecting neuronal M2 muscarinic receptors in antigen-challenged guinea pigs.. J Clin Invest.

[42] Jacoby DB, Gleich GJ, Fryer AD (1993). Human eosinophil major basic protein is an endogenous allosteric antagonist at the inhibitory muscarinic M2 receptor.. J Clin Invest.

[43] Elbon CL, Jacoby DB, Fryer AD (1995). Pretreatment with an antibody to interleukin-5 prevents loss of pulmonary M2 muscarinic receptor function in antigen-challenged guinea pigs.. Am J Respir Cell Mol Biol.

[44] Proskocil BJ, Bruun DA, Lorton JK, Blensly KC, Jacoby DB (2008). Antigen sensitization influences organophosphorus pesticide-induced airway hyperreactivity.. Environ Health Perspect.

[45] Lotti M, Spencer PS, Schaumburg HH, Ludolph AC (2000). Organophosphorus compounds.. Experimental and Clinical Neurotoxicology. 2nd ed.

[46] Pope CN (1999). Organophosphorus pesticides: do they all have the same mechanism of toxicity?. J Toxicol Environ Health B Crit Rev.

[47] Jacoby DB, Xiao HQ, Lee NH, Chan-Li Y, Fryer AD (1998). Virus- and interferon-induced loss of inhibitory M2 muscarinic receptor function and gene expression in cultured airway parasympathetic neurons.. J Clin Invest.

[48] Jacoby DB, Yost BL, Kumaravel B, Chan-Li Y, Xiao HQ (2001). Glucocorticoid treatment increases inhibitory m(2) muscarinic receptor expression and function in the airways.. Am J Respir Cell Mol Biol.

[49] Moreno L, Jacoby DB, Fryer AD (2003). Dexamethasone prevents virus-induced hyperresponsiveness via multiple mechanisms.. Am J Physiol Lung Cell Mol Physiol.

[50] Lein PJ, Fryer AD, H D, Squire LR (2009). Cell Culture: Autonomic and Enteric Neurons.. Encyclopedia of Neuroscience.

[51] Nie Z, Jacoby DB, Fryer AD (2009). Etanercept prevents airway hyperresponsiveness by protecting neuronal M2 muscarinic receptors in antigen-challenged guinea pigs.. Br J Pharmacol.

[52] Schopfer LM, Voelker T, Bartels CF, Thompson CM, Lockridge O (2005). Reaction kinetics of biotinylated organophosphorus toxicant, FP-biotin, with human acetylcholinesterase and human butyrylcholinesterase.. Chem Res Toxicol.

[53] Peeples ES, Schopfer LM, Duysen EG, Spaulding R, Voelker T (2005). Albumin, a new biomarker of organophosphorus toxicant exposure, identified by mass spectrometry.. Toxicol Sci.

[54] Ecobichon DJ, Klaassen CD (2001). Toxic effects of pesticides.. Casarett and Doull's Toxicology: The Basic Science of Poisons.

[55] Ehrich M, Intropido L, Costa LG (1994). Interaction of organophosphorus compounds with muscarinic receptors in SH-SY5Y human neuroblastoma cells.. J Toxicol Environ Health.

[56] Fryer AD, Jacoby DB (1998). Muscarinic receptors and control of airway smooth muscle.. Am J Respir Crit Care Med.

[57] Jett DA, Fernando JC, Eldefrawi ME, Eldefrawi AT (1994). Differential regulation of muscarinic receptor subtypes in rat brain regions by repeated injections of parathion.. Toxicol Lett.

[58] Ward TR, Mundy WR (1996). Organophosphorus compounds preferentially affect second messenger systems coupled to M2/M4 receptors in rat frontal cortex.. Brain Res Bull.

[59] Billington CK, Penn RB (2002). m3 muscarinic acetylcholine receptor regulation in the airway.. Am J Respir Cell Mol Biol.

[60] Savolainen KM, Hirvonen MR (1992). Second messengers in cholinergic-induced convulsions and neuronal injury..

[61] Kilbinger H, Wessler I (1980). Inhibition by acetylcholine of the stimulation-evoked release of [3H]acetylcholine from the guinea-pig myenteric plexus.. Neuroscience.

[62] Kilbinger H, Dietrich C, von Bardeleben RS (1993). Functional relevance of presynaptic muscarinic autoreceptors.. J Physiol Paris.

[63] Udarbe Zamora EM, Liu J, Pope CN (2008). Effects of chlorpyrifos oxon on M2 muscarinic receptor internalization in different cell types.. J Toxicol Environ Health A.

[64] Bomser JA, Casida JE (2001). Diethylphosphorylation of rat cardiac M2 muscarinic receptor by chlorpyrifos oxon in vitro.. Toxicol Lett.

[65] Bernard V, Decossas M, Liste I, Bloch B (2006). Intraneuronal trafficking of G-protein-coupled receptors in vivo.. Trends Neurosci.

[66] Fryer AD, el-Fakahany EE (1989). An endogenous factor induces heterogeneity of binding sites of selective muscarinic receptor antagonists in rat heart.. Membr Biochem.

[67] Koenig JA, Edwardson JM (1996). Intracellular trafficking of the muscarinic acetylcholine receptor: importance of subtype and cell type.. Mol Pharmacol.

[68] Nelson CP, Gupta P, Napier CM, Nahorski SR, Challiss RA (2004). Functional selectivity of muscarinic receptor antagonists for inhibition of M3-mediated phosphoinositide responses in guinea pig urinary bladder and submandibular salivary gland.. J Pharmacol Exp Ther.

[69] Dabisch PA, To F, Kerut EK, Horsmon MS, Mioduszewski RJ (2007). Multiple exposures to sarin vapor result in parasympathetic dysfunction in the eye but not the heart.. Toxicol Sci.

[70] Sun T, Ma T, Ho IK (2003). Differential modulation of muscarinic receptors in the rat brain by repeated exposure to methyl parathion.. J Toxicol Sci.

[71] Yagle K, Costa LG (1996). Effects of organophosphate exposure on muscarinic acetylcholine receptor subtype mRNA levels in the adult rat.. Neurotoxicology.

[72] Finkelman FD, Boyce JA, Vercelli D, Rothenberg ME (2009). Key advances in mechanisms of asthma, allergy, and immunology in 2009.. J Allergy Clin Immunol.

[73] Lee AM, Fryer AD, van Rooijen N, Jacoby DB (2004). Role of macrophages in virus-induced airway hyperresponsiveness and neuronal M2 muscarinic receptor dysfunction.. Am J Physiol Lung Cell Mol Physiol.

[74] Yoon HK, Cho HY, Kleeberger SR (2007). Protective role of matrix metalloproteinase-9 in ozone-induced airway inflammation.. Environ Health Perspect.

[75] Rodgers KE, Ellefson DD (1990). Modulation of respiratory burst activity and mitogenic response of human peripheral blood mononuclear cells and murine splenocytes and peritoneal cells by malathion.. Fundam Appl Toxicol.

[76] Rodgers KE, Ellefson DD (1990). Modulation of macrophage protease activity by acute administration of O,O,S trimethyl phosphorothioate.. Agents Actions.

[77] Rodgers K, Xiong S (1997). Effect of administration of malathion for 14 days on macrophage function and mast cell degranulation.. Fundam Appl Toxicol.

[78] Flipo D, Bernier J, Girard D, Krzystyniak K, Fournier M (1992). Combined effects of selected insecticides on humoral immune response in mice.. Int J Immunopharmacol.

[79] Rodgers K, Xiong S (1997). Effect of acute administration of malathion by oral and dermal routes on serum histamine levels.. Int J Immunopharmacol.

[80] Rodgers K, Ellefson D (1992). Mechanism of the modulation of murine peritoneal cell function and mast cell degranulation by low doses of malathion.. Agents Actions.

[81] Xiong S, Rodgers K (1997). Effects of malathion metabolites on degranulation of and mediator release by human and rat basophilic cells.. J Toxicol Environ Health.

[82] Rodgers K, Xiong S (1997). Contributions of inflammatory mast cell mediators to alterations in macrophage function after malathion administration.. Int J Immunopharmacol.

[83] Levy A, Chapman S, Cohen G, Raveh L, Rabinovitz I (2004). Protection and inflammatory markers following exposure of guinea pigs to sarin vapour: comparative efficacy of three oximes.. J Appl Toxicol.

[84] Pena-Philippides JC, Razani-Boroujerdi S, Singh SP, Langley RJ, Mishra NC (2007). Long- and short-term changes in the neuroimmune-endocrine parameters following inhalation exposures of F344 rats to low-dose sarin.. Toxicol Sci.

[85] Hermanowicz A, Kossman S (1984). Neutrophil function and infectious disease in workers occupationally exposed to phosphoorganic pesticides: role of mononuclear-derived chemotactic factor for neutrophils.. Clin Immunol Immunopathol.

[86] Duramad P, Tager IB, Leikauf J, Eskenazi B, Holland NT (2006). Expression of Th1/Th2 cytokines in human blood after in vitro treatment with chlorpyrifos, and its metabolites, in combination with endotoxin LPS and allergen Der p1.. J Appl Toxicol.

[87] Verhein KC, Jacoby DB, Fryer AD (2008). IL-1 receptors mediate persistent, but not acute, airway hyperreactivity to ozone in guinea pigs.. Am J Respir Cell Mol Biol.

[88] Fryer AD, Jacoby DB (1992). Function of pulmonary M2 muscarinic receptors in antigen-challenged guinea pigs is restored by heparin and poly-L-glutamate.. J Clin Invest.

[89] Canning BJ (2003). Modeling asthma and COPD in animals: a pointless exercise?. Curr Opin Pharmacol.

[90] Smolen A, Smolen TN, Oh EI, Collins AC (1986). A strain comparison of physiological and locomotor responses of mice to diisopropylfluorosphosphate.. Pharmacol Biochem Behav.

[91] Smolen A, Smolen TN, Wehner JM, Collins AC (1985). Genetically determined differences in acute responses to diisopropylfluorophosphate.. Pharmacol Biochem Behav.

[92] Costa LG (2006). Current issues in organophosphate toxicology.. Clin Chim Acta.

[93] Coulson FR, Jacoby DB, Fryer AD (2002). Increased function of inhibitory neuronal M2 muscarinic receptors in trachea and ileum of diabetic rats.. Br J Pharmacol.

[94] Ellman GL, Courtney KD, Andres V, Featherstone RM (1961). A new and rapid colorimetric determination of acetylcholinesterase activity.. Biochem Pharmacol.

[95] Higgins D, Lein P, Osterhout D, Johnson M, Banker G, Goslin K (1991). Tissue culture of mammalian autonomic neurons.. Culturing Nerve Cells 1 ed.

[96] Yang D, Howard A, Bruun D, Ajua-Alemanj M, Pickart C (2008). Chlorpyrifos and chlorpyrifos-oxon inhibit axonal growth by interfering with the morphogenic activity of acetylcholinesterase.. Toxicol Appl Pharmacol.

[97] Schlador ML, Grubbs RD, Nathanson NM (2000). Multiple topological domains mediate subtype-specific internalization of the M2 muscarinic acetylcholine receptor.. J Biol Chem.

[98] Dhein S, van Koppen CJ, Brodde OE (2001). Muscarinic receptors in the mammalian heart.. Pharmacol Res.

